# Predictive Factors in the Appearance and Evolution of Squamous Cell Carcinomas of the Oral Cavity

**DOI:** 10.3390/medicina58050570

**Published:** 2022-04-21

**Authors:** Alexandra Carp, Andrei Nicolau, Mihaela Moscalu, Eugenia Popescu

**Affiliations:** 1 Department of Oral and Maxillofacial Surgery, Grigore T. Popa University of Medicine and Pharmacy, 700115 Iasi, Romania; alexandra.i.carp@umfiasi.ro (A.C.); eugenia.popescu@umfiasi.ro (E.P.); 2 Department of Preventive Medicine and Interdisciplinarity, Division of Informatics and Medical Statistics, Grigore T. Popa University of Medicine and Pharmacy, 700115 Iasi, Romania

**Keywords:** oral squamous cell carcinoma, risk factors, screening, early diagnosis

## Abstract

*Background*: Oral squamous cell carcinoma (OSCC) registered an alarming fall in the average age of individuals diagnosed in the last decade. *Objectives:* The aim of our study is to assess the main risk factors for OSCC specific to Romania and to identify patients at risk for this pathology. The purpose is to implement in the future a screening and early diagnosis program for OSCC in our country. *Materials and Methods*: A ten-year case-control study was conducted on patients selected from “St. Spiridon” Hospital-Iaşi, Romania. The study contained 1780 individuals diagnosed with oral squamous cell carcinoma. *Results*: For the patients under 46 years old: APC = −2.8 percent (95% CI: −24.4 to −7.1; *p* = 0.0012), with the observed rate of 30.18 percent. The incidence increased in patients aged 46 to 49 years (APC = 9.6%; 95% CI: 6.7 to −10.4; *p* = 0.0081). For the age group 49 to 64 years old: APC = −2.4 percent (95% CI: −5.3 to −1.6, *p* = 0.1239). For the age group 64–74: APC = −4.6, (95% CI: 1.4 to 6.9, *p* = 0.0108). The incidence of incidents was lower in the age group 74–80 (*p* = 0.0025). For the age group 80–91: APC = 8.1 (95% CI: 6.4 to 14.2, *p* = 0.0024), with the incidence of cases: APC = 8.1 (95% CI: 6.4 to 14.2, *p* = 0.0024). Univariate analysis revealed a substantially higher risk of developing oral carcinoma in males (OR = 4.43; CI: 3.84 to 5.80). Age above 60, cigarette usage and alcohol abuse are significant risk factors for OSCC. Patients with lymph node dissemination, ulcero-vegetant form, stages II and IV, whose therapeutic approach consisted of radiotherapy and chemotherapy or radiotherapy only had a worse rate of survival at 24 months post-therapy. *Conclusions*: Our study highlights the increase in the incidence of OSCC in Romania during the research period, the decrease in the average age of diagnosed patients, as well as the degree to which the studied population is exposed to the main risk factors specific to this geographical area.

## 1. Introduction

The latest statistics provided by GLOBOCAN for oral cavity and lip cancer ranked this tumor as the sixth most common malignancy worldwide, with 377,713 new cases and 177,757 deaths in 2020. The highest rate was encountered in Central and South Asia followed by East Asia [1].

The environmental factors that influence the development of OSCC are diverse and significantly different in their global prevalence, due to the particular customs of the territory.

Although studies have been conducted on OSCC, they are more frequent in countries where local habits induce the development of the neoplasia (consumption of smokeless tobacco products, reverse smoking, pipe smoking, marijuana smoking, e.g., [2]).

These habits are rarer in our geographical area, and therefore, the prevalence of OSCC is lower in Eastern Europe than in Asian countries.

In Europe, this malignancy ranks 11st in terms of mortality rate, with a cumulative annual incidence of 18.2 in men and 4.9 in women [3].

In over 90% of instances, smoking and chronic alcohol intake are regarded the key etiological factors implicated in the development of oral cancer, with a synergistic effect on the mucosa of the oral cavity [4].

Smokers have a threefold increased chance of developing OSCC compared to non-smokers. When compared to non-smokers, it reduces by 35% among persons who quit smoking at least 4 years ago. It is, however, identical for people who have not smoked in at least 20 years and for those who have never smoked [5].

Romania reported 3320 new cases of OSCC in 2015, and the number of cases increased in subsequent years [6].

Although the incidence of OSCC has increased in recent decades, Romania presents a minor number of studies on prevalence, risk groups for patients and main risk factors for this malignancy. In addition, no screening or early diagnosis program has been implemented in our country up to the present.

Smoking and chronic alcohol consumption are considered the main etiological factors involved in the development of OSCC in our country. There are also other factors listed such as HPV infection and prolonged exposure to ultraviolet radiation (for lip cancer), age (over 50 years), sex (male), poor oral hygiene, and area of origin (rural) [7].

OSCC is typically characterized by a late diagnosis, various morbidities, and major aesthetic changes, with a disfiguring aspect in advanced stages [8]. This neoplasm is also frequently discovered in a metastatic stage. The prognosis is substantially worse in this situation due to the primary tumor’s rapid growth, which damages the deep tissues [9,10].

Surgery is the therapy of choice for patients with squamous cell carcinoma of the oral mucosa and is usually followed by adjuvant treatment, especially when lymphoganglionary spread is present (radiochemotherapy).

Squamous cell carcinoma is the most common histological form of primary tumor, accounting for 94% of cases. Adenocarcinomas, cystic adenoid carcinomas, mucoepidermoid carcinomas, and lymphomas make up the remaining percentages. Furthermore, tumor differentiation has been proven to make well-differentiated cancers less aggressive than poorly differentiated tumors [11].

Every patient with oral cancer should be treated by a multidisciplinary team with expertise in the treatment of head and neck malignancies, and any suspicious lesion should be referred to an oral and maxillofacial surgeon or an oral medicine specialist as soon as possible [12].

The aim of the study was to conduct a complete evaluation of the etiological factors implicated in the occurrence of OSCC, as well as to implement effective population screening and early diagnosis procedures for patients at risk.

## 2. Materials and Methods

A ten-year case-control study (from 1 January 2010 to 31 December 2019) was conducted. The patients were selected from the Oral and Maxillofacial Clinic at the “St. Spiridon” Hospital in Iaşi, Romania. They were divided into two groups.

Of all cancers located in the oral cavity, in our study, we included only patients who were diagnosed with OSCC (oral mucosal cancer).

The Ethical Committee of Iasi’s Clinical Hospital “St. Spiridon” gave its approval to the study (No. 24225/November 2020). Following the 2013 revision of the Helsinki Declaration, all participants gave their informed consent.

For TNM classification of the OSCC, we used the (2011) TNM Classification Encyclopedia of Cancer Springer [13].

SPSS v.25 was used to conduct the statistical analysis of the variables of interest (IMB Corporation, New Orchard Road Armonk, New York, USA). Depending on the normal distribution of the values, we calculated the means and standard deviation (mean SD) or the median and interquartile range (IQR) for continuous variables. The Kruskal–Wallis test for continuous variables was used to make comparisons between the statistical groupings. The Levene test was performed to determine whether the variances were homogeneous.

We examined frequencies (absolute and relative percent) and performed group comparisons based on the results of non-parametric tests for qualitative variables (Pearson chi-square). Overall survival (OS) and event-free survival (EFS) were calculated using the Kaplan–Meier technique (EFS). To make comparisons, we used the log-rank test. The Joinpoint regression model was used to examine the relationship between age and oral cancer incidence.

The junctions at which the incidence of oral cancer began to increase or decrease, as measured across all age groups, were found. To this goal, we created a univariable regression of a connection point at which the age-incidence association was investigated. For each effect estimate, the annual percentage change (APC) and the related 95 percent confidence interval (CI) were determined. The statistical significance level was established at *p* < 0.05.

## 3. Results

**Figure 1:**
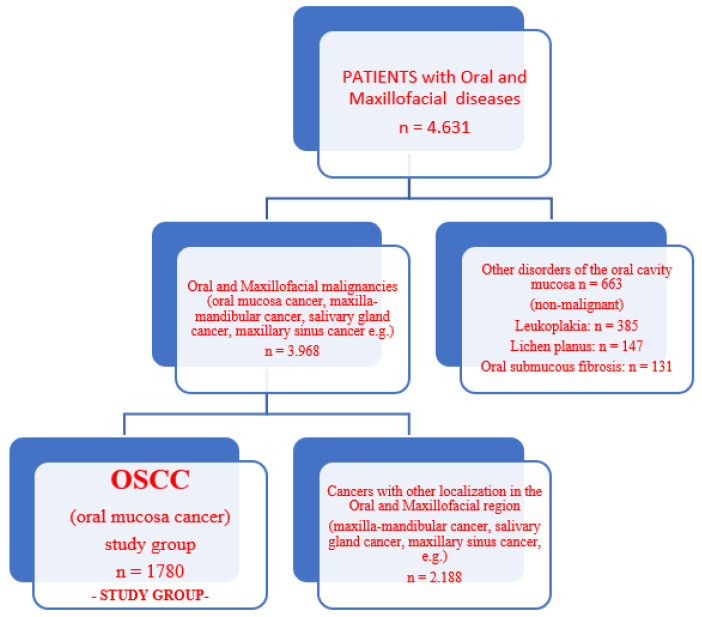
Flow chart for patient selection.

The study comprised 1780 individuals diagnosed with oral squamous cell carcinoma, ranging in age from 24 to 83 years (median age = 60.9 ± 11.6 SD). Anatomopathological confirmation of the patient’s diagnosis was obtained. There were 663 patients in the control group who had other non-oncological oral diseases (Figure 1).

The patients in the research had surgical treatment, which is the preferred treatment for oral squamous cell carcinomas.

The incisional biopsy, which took place before the surgical stage of treatment, confirmed the presence of cancer and the degree of differentiation.

The procedure entailed totally removing the tumor (within the limits of oncological safety) as well as the cervical lymph nodes (if the lymph nodes are in the metastatic stage).

The post-operator defect was plastically reconstructed using marginal sutures, grafts, or flaps, depending on its size (local, loco-regional, or free transferred).

The patient got adjuvant treatment (radio/chemotherapy) after discharge from the clinic, based on the anatomopathological findings. If extracapsular lymph node extension was discovered, chemotherapy was administered. Cisplatin or epidermal growth factor inhibitors like Cetuximab were part of the treatment plan.

The patient’s progress was monitored monthly for the first year after surgery to detect any possible oral cancer recurrence, then at 15 months, 18 months, and 24 months.

The risk of tumor recurrence, which is higher in the first two years following treatment, has been thoroughly assessed. As a result, our research looked at the patient’s survival rate after 24 months—the absence of symptoms and the lack of imagistic proof of the tumor’s presence after 5 years suggested that the patient was healed (cancer free).

All of the cases in our study were followed up on clinically and/or radiologically. Because of the severe morbidity associated with the procedure, speech and swallowing rehabilitation, as well as the retention and repair of the remaining teeth, were sought. Psychological counseling was provided to the patients.

Computed tomography (CT), magnetic resonance imaging (MRI), ultrasonography (US), and positron emission tomography (PET) were used in the evaluation for staging. Oral cancer was assessed using CT scans of the head and neck anatomical region, as well as the thoracic region, for potential lymph node, brain, and lung metastases.

The primary risk factors for oral squamous cell carcinomas were investigated. Tobacco smoking, alcohol drinking, and poor oral hygiene were considered as four health-related behaviors linked to oral cancer.

The interval between diagnosis and the last follow-up—which signals healing or the first important event, such as recurrence or death—is known as event-free survival (EFS).

The time span from diagnosis and the last follow-up or death was defined as overall survival (Srvvng).

After executing a curative treatment for the previously treated neoplasia, tumor recurrence was described as the reappearance of local clinical indications of the disease or the emergence of a new tumor in a different place.

It is also worth noting that patient tracking has been going on for the past 24 months.

### 3.1. Statistical Analysis

During our research, 1780 patients (44.9%) of the 3968 patients with oral and maxillofacial carcinomas admitted to the Oral and Maxillofacial Surgery Clinic had carcinomas of the lips and oral cavity (squamous cell carcinomas of the oral mucosa).

According to clinic data, there was an increase in the incidence of oral carcinomas from 2010 to 2019. The largest number of diagnosed cases was recorded in 2019, accounting for 7.08 percent of the total number of studied cases.

Male cases with oral carcinomas (83.8 percent) had a substantially higher incidence (χ^2^ = 5.48, *p* < 0.001) in the study group. There was also a substantially greater frequency (χ^2^ = 38.22, *p* = 0.00251) of cigarette users (82.7%) and chronic alcohol users (72.7%, *p* < 0.001) (Table 1). In the study group, poor oral hygiene was linked to 31.7 percent of patients (*p* = 0.004).

Comorbidities such as endocrine pathologies (hypo/hyperthyroidism), cardiovascular illnesses (myocardial infarction), and rheumatological diseases (rheumatoid arthritis) were investigated in relation to the occurrence of carcinomas of the oral mucosa.

Localization at the level of the tongue (23.15 percent) was the most common, whereas localization at the level of the intermaxillary commissure with jugal extension or towards the lateral wall of the pharynx had the lowest frequency (3.03 percent).

Ulcero-vegetant lesions accounted for 23.6 percent of all lesions, followed by ulcero-destructive lesions (22.3 percent) (Table 1). After a clinical examination 74.6 percent of hospitalized patients were diagnosed with metastatic lymphadenopathy, while 25.4 percent had no evidence of lymph node spread. At the time of hospitalization, the ratio was 2.9 in favor of the occurrence of metastatic lymphadenopathy (Table 1).

In impoverished or developing nations, where access to the dentist/general practitioner is limited and health education is ineffective, the diagnosis is verified more commonly in advanced stages III (27.4%) and IV (23.7%). In our research, we came up with a similar finding.

Various reference thresholds for estimating the risk of this pathology’s incidence have been proposed in the literature. An analysis of the Jointpoint regression model was performed in this context to determine the relationship between age and the incidence of carcinomas of the oral mucosa. There was a non-linear relationship between patients and the incidence of oral cancer (Table 1). There have been five junction sites found where the incidence of squamous cell carcinoma has altered with age. For each effect estimation, the annual percentage change (year of age) (APC) with the 95% confidence interval (CI) was determined.

There was a significant inverse association between age (under 46 years old) and the incidence of oral carcinomas (APC—annual percentage change = −2.8 percent; 95% CI: −24.4 to −7.1; *p* = 0.0012), with the observed rate of 30.18 percent (modeled rate: 33.7 percent) at age 46 (Table 1, Figure 1).

The incidence of oral carcinomas increased considerably in patients aged 46 to 49 years (APC = 9.6%; 95% CI: 6.7 to −10.4; *p* = 0.0081) (Table 2, Figure 1). For the next age group, 49 to 64 years old, regressive analysis revealed a decreasing trend in incidence, but it was minor. This resulted in an APC value of −2.4 percent (95% CI: −5.3 to −1.6, *p* = 0.1239), demonstrating a non-significant negative regression of case incidence in the 49–64-year-old age group (Table 2, Figure 1).

A considerable positive linear regression of the incidence of carcinogenic cases of the oral mucosa is seen in the group of people aged 64 to 74 years. As a result, the incidence of cases rises with increasing age in this interval (APC = −4.6, 95% CI: 1.4 to 6.9, *p* = 0.0108). At 74 years old, the observed gross rate was 50.8 percent, while the modeled rate was 48.1 percent. The incidence was significantly lower in the 74–80-year-old group (*p* = 0.0025).

A substantial rise in the incidence of cases was detected in the group of people aged 80 to 91 years old, with APC = 8.1 (95% CI: 6.4 to 14.2, *p* = 0.0024) being recorded. This finding reveals a considerable increase in the number of cases among people aged 81 to 90. (Table 2, Figure 1).

We used the age of 60 as a reference threshold based on the results of the examination of the link between the age of the patients and the occurrence of cases in this study. We chose the age of 60 as a benchmark for our study because most of the patients who were admitted in our clinic were older than this value, smoking being a habit that the population of our country practices starting at more advanced stages of life in comparison to other countries (India, Taiwan).

Univariate and multivariate analysis were used to look for predicting indicators for the appearance of oral carcinomas (Table 3).

When compared to the risk of developing oral cancer in females (OR = 4.43; CI: 3.84 to 5.80), univariate analysis revealed a substantially higher risk of developing oral carcinoma in males (OR = 4.43; CI: 3.84 to 5.80) (Table 3). Additionally, age above 60 (OR = 1.95; CI: 1.38 to 2.59), cigarette usage (OR = 2.46; CI: 1.15 to 3.63), and alcohol abuse (OR = 2.79; CI: 1.27 to 4.05) are also significant risk factors for oral carcinomas.

The “ENTER” model was used for the multivariate analysis of risk factors, in which all independent variables (risk factors in the development of oral carcinomas) were incorporated in one stage. The applied model was tested to see if it had any predictive power.

The findings show that the model can accurately evaluate a large number of examples (χ^2^ = 26.4, *p* = 0.001, −2LL = 42.6). The model’s calibration results for the studied data (Hosmer–Lemeshow test) show that the predicted frequency of cases with oral carcinomas is not statistically different from the model’s estimate (*p* = 0.472) (Table 3).

### 3.2. Survival Analysis

The survival rate (SR) differed significantly depending on the type of treatment (*p* = 0.0009) (Table 4). The best survival rates were observed in the study group when surgical intervention was followed by radiotherapy, whether or not it was combined with chemotherapy.

As a result, at the end of the 24-month follow-up, the survival rate for cases treated with surgery followed by radiotherapy was 83.3%. When chemotherapy was added, the percentage dropped to 66.67%.

Patients whose therapeutic approach consisted of radiotherapy and chemotherapy (SR 24 months = 54.5 percent) or radiotherapy only (SR 24 months = 51.84 percent) had a worse rate of survival at 24 months post-therapy (Table 4). 

The ability to assess the risk of an event has a significant prognostic value. Patients over 60 years old (24.6 percent), male patients (77.5%), and patients with ulcero-vegetant forms also had higher rates of EFS at 24 months of follow-up. The findings show that lymph node dissemination, together with the ulcero-vegetant form, stages II and IV, is a high-risk factor that affects EFS rates (Table 5). The last table shows the EFS for the factors studied in individuals with carcinomas of the oral cavity.

## 4. Discussion

Oral cancer accounts for over 85% of head and neck cancers—brain cancer is not included in this statistic—and registers a high mortality rate [4].

Worldwide, in terms of the number of deaths due to OSCC, Romania ranks 20st position [14].

Smoking is a major risk factor, and according to the Global Burden of Disease, more than 8,000,000 individuals died in 2017 as a result of smoking-related disorders. As stated by the same survey, one out of every five persons, usually men, smokes (35 percent). Women account for only 6% of smokers [15].

In 2019, a large study was undertaken on a global scale that contained substantial data on the number of smokers, the number of cigarettes smoked per day, the type of cigarettes used and fatalities from smoking-related diseases, and our country was included in the study.

In Romania, roughly 30% of the population smokes, with an average of 20 cigarettes per day. Cancer was responsible for one out of every five deaths worldwide in 2016, according to the World Health Organization. In Romania, the percentage is between 20% and 30% [16].

When compared to non-smokers who have never been exposed to cigarette smoke, passive smoking raises the risk of acquiring mouth cancer by 87 percent. The oral mucosa is exposed to free radicals of oxygen and nitrogen as a result of tobacco use. Precancerous and cancerous lesions have high quantities of free radicals [11].

Although smoking is the most serious risk factor, other behaviors unique to different parts of the world have been emphasized. Snuffing (in Sweden, United States), using mishri and betel nut in India and chewing Iq’mik (in AK, USA) are among the most well-known. Oral cancer is more common in such places as a result of them. Furthermore, according to certain professional studies, drinking maté tea in Latin America doubles the risk of mouth cancer due to the high temperature and carcinogenic compounds present [17].

Alcohol use, along with smoking, is a significant risk factor in the development of oral carcinomas. In 2016, 2.4 billion people consumed alcohol. In Romania, men consume alcohol at a rate of 80%, while women use alcohol at a rate of 60%. At the same time, studies have shown that the pathology associated with chronic alcohol consumption has resulted in a significant number of deaths in people over 50 (28% of men and 19% of women) [18].

According to specialized studies, chronic drinkers account for seven out of ten people diagnosed with oral cancer. Alcohol increases the permeability of the oral mucosa, causing the lipid component of the epithelium to dissolve over time, resulting in epithelial atrophy and interfering with DNA synthesis and repair. Simultaneously, it causes genotoxicity and mutagenic effects, reduces salivary secretion, impairs the liver’s ability to remove toxins and potentially carcinogenic substances, and lowers immunity, increasing the risk of infection and neoplasia [11].

The overall amount of ethanol in alcoholic beverages plays a significant role in the initiation of the tumor pathological process. The risk of developing cancer of the oral mucosa increases tenfold in chronic alcohol users compared to those who drink only occasionally or not at all. At the same time, poor dental hygiene is another risk factor for the development of oral cancer, which increases the production of acetaldehyde [19].

The annual incidence of malignant transformation of premalignant lesions varies between 0.13 and 2.2 percent [20]. Candida Albicans’ ability to form biofilm, hydrolytic enzymes, and metabolize alcohol to make acetaldehyde, as well as its ability to contribute to the development of OSCC, have all been linked in recent studies [21].

Genetic factors also play a role in the appearance of oral carcinomas. Tumor suppressor genes (p53), oncogenes (Ras), proto-oncogenes (Myc) and genes that influence normal cellular processes (GSTM1, EIF3E) have all been linked to the disease. Tumor activity is influenced by heterozygotic loss, chromosome segregation, telomere stabilization, DNA repair, and genomic copied number [22].

Immunocompromised people with Kaposi’s sarcoma, lymphoma, or HIV are more prone to acquire cancer, according to research.

Prolonged exposure to solar radiation, which is primarily owing to patients’ employment conditions, is another risk factor for the development of lip carcinomas. Other long-term exposures to harmful substances are also implicated in the event of malignant proliferation. Asbestos, sulfur dioxide, and pesticides have all been linked to oral cancer, according to research.

Laboratory studies have repeatedly proven a relationship between diet and OSCC, demonstrating that a poor consumption of fruits and vegetables increases the risk of malignancy [23].

The histological type represents essential information connected to oral carcinomas, as well as the risk factors involved in the occurrence of the malignant process. As a result, squamous carcinomas account for over 90% of oral malignancies, with varied degrees of histological differentiation and a proclivity for lymph nodes [11].

According to a study conducted in Brazil, men account for 67 percent of the 346 patients diagnosed with oral squamous cell carcinoma, with an average age of roughly 62 years (the average age of women being 72 years).

The tongue (37 percent), alveolar ridge (20 percent), and the ventral face of the tongue/the floor of the mouth were the most prevalent places (19 percent). The tumor measured roughly 3.4 cm in diameter on average. Male patients have confirmed the link between smoking and alcohol usage more frequently than female patients. 

The study confirmed well-differentiated tumors in 27% of cases, moderately differentiated tumors in 40% of cases, and poorly differentiated tumors in 21% of cases. Minimally invasive squamous cell carcinomas were found in 26 patients (7.5%) [24].

Our findings are consistent with data from the international scientific literature. 300,000 new cases of oral carcinomas were diagnosed over the world in 2012. From 1970 to the present, the incidence has climbed by 92 percent in England [11].

Romania is ranked fifth in Europe in terms of the incidence of squamous cell carcinomas of the oral mucosa, with an annual increase of 12.88 percent [25].

The highest number of individuals with cancer of the oral mucosa was documented in our study in 2019 (258 cases).

Oral cancer had the highest incidence rate (21%) in this study, with a 2/1 ratio between men (64 percent—poor socioeconomic status in 59 percent of cases) and women. The 45–64-year-old age group was the most affected by oropharyngeal cancer.

Long-term sun exposure is a major risk factor for developing lip cancer. The risk of developing lip cancer increases dramatically when you smoke. The main risk factors for oral carcinomas include smoking and alcohol consumption. 

Between 2010 and 2019, rural areas were accounted for the majority of patients admitted for oral carcinomas (780 cases, or 43.82 percent). This result is explained by the fact that agriculture is the main occupation for these residents. Consequently, they were constantly exposed to ultraviolet radiation, without using sunscreens. In addition, most of the patients were also subjected to other risk factors for OSCC such as chronic alcohol consumption and heavy smoking.

In our study, 1145 patients (64.33 percent) acknowledged smoking; 328 patients (18.4%) were ex-smokers, and 230 patients (45.63%) smoke more than a pack of cigarettes every day. For 208 patients, tobacco use has been ongoing for more than 30 years (41.26 percent).

Seven hundred and seventy-eight patients (43.71 percent) were chronic alcoholics, while 507 patients (28.48 percent) were occasional alcoholics.

The combination of smoking and alcohol consumption nearly triples the risk of oral and pharyngeal cancer [26]; the risk for the smoker increases with the amount of alcohol consumed, as ethanol has an hyperpermeabilizing effect on oral epithelial cells in response to tobacco carcinogens [27].

Numerous studies [28,29] have been published on the link between oral hygiene and the development of OSCC. They consider factors such as the oral hygiene index, the presence of halitosis, the number of missing teeth, brushing techniques used, the usage of mouthwash and so on. As a result, according to a study conducted in India on 337 people diagnosed with oral cancer, around 79 percent of them have poor oral hygiene [30]. Individuals’ cleanliness habits must be identified, as well as their motivation to maintain the greatest dental-periodontal status, as part of the screening required to get an early diagnosis of malignant tumor proliferation.

Poor oral hygiene was found to be present in up to 78.18 percent of patients in our study. Patients with periodontal disease have a 2.6-fold increased risk of developing oral mucosa carcinomas [31]. 

In the current study, the largest number of patients with oral mucosa cancer was observed in 2019 (258 instances), with men having a much greater frequency of cases (83.82 percent) than women (16.18 percent).

This predilection for the male sex can be found in a variety of other statistics, such as one from a UK study of 7.591 new oral cancer cases conducted in 2013. Men made up 5.103 (67%) while women made up 2.488 (33%) of the total. Men outnumbered women by a factor of 2.05 [32].

A study conducted in London (1998–2009) looked at standardized yearly incidence rates for malignancies with diverse locations. Oral and oropharyngeal cancer had the highest incidence (21%), with a 2/1 ratio between men and women, according to this study [33]. 

In terms of patient age, the 51–60 age group had the highest incidence rate in our research (569 patients). Between 2011 and 2013, 45 percent of patients diagnosed with oral cancer in England were over 65 years old, according to a study. According to published data, the number of illnesses is beginning to rise significantly among those aged 40 to 44, with the category 60–64 having the highest number of instances [32].

Over the course of 12 years (2000–2012), a large-scale study involving 51,116 patients in Brazil yielded relevant data on oral malignant tumors. Oral cancer was identified in advanced stages (III and IV) in the majority of age groups, with the majority of cases occurring in those aged 56 to 64. (22,731 patients; 44.5 percent). This is supported by long-term exposure to risk factors (alcohol consumption, smoking). Only 6487 patients (12.7%) were found to be in stages I and II [34].

Between 2010 and 2019, rural areas accounted for the majority of hospitalized patients with oral cancer (1000 patients—56.2 percent). Solar radiation, and implicitly extended exposure to it, is a primary risk factor for the incidence of oral carcinomas in rural settings. We identified 8 cases (0.9 percent) of lesions with the potential for malignant transformation that occurred on leukoplakia.

Tongue cancer was shown to be the most common type of cancer (412 cases, or 23.15 percent), followed by pelvilingual localization (374 cases, or 21.01 percent) and lip cancer (362 cases—20.34 percent). 

The gingival-alveolar area (187 patients—10.51 percent), the intermaxillary commissure expanded in the jugal region or to the lateral wall of the pharynx (54 patients—3.03 percent), and the palatal veil (54 patients—3.03 percent) had the fewest cases (4.44 percent).

These findings are similar to those described in Europe, where the tongue (40 percent of patients) is the most affected location, followed by pelvilingual localization. The gingival–alveolar ridge and the posterior palate have a reduced number of instances. Oral cancer most commonly affects the tongue in men, followed by the posterior palate, while it affects the palate, gums, and gingival-alveolar junction in women [32].

In terms of histological differentiation, the majority of our patients (471 instances, or 58.07 percent) had well-differentiated squamous cell carcinomas. The G1 stage (25 percent undifferentiated cells) was the most common, according to Broder’s classification [35].

We discovered that 1321 patients (74.21 percent) had lymph node invasion since hospitalization when we correlated the degree of histological differentiation with the occurrence of loco-regional metastatic lymphadenopathy. They were in the advanced stages of the condition when they went to the professional consultation.

The limitations of our study consist in the fact that we have not analyzed a larger number of risk factors for OSCC, but we aim to design larger confirmatory studies in the future. 

## 5. Conclusions

Our study highlights the high incidence and prevalence of OSCC in Romania and the distribution of the patients according to specific demographic parameters. 

The statistical analysis also documents the main risk factors for OSCC that are encountered in our geographical area. By adding the evaluation of the histopathological form and cell differentiation, we present a complex documentation of OSCC in Romania.

A multidisciplinary strategy, including health education, tobacco and alcohol control, and surgical treatment in the preliminary stages of the disease, is essential for reducing the number of cases of oral squamous cell carcinoma, which is highly aggressive and has a high fatality rate. 

The data obtained in our study can lead to the identification of effective screening methods designed to aid early diagnosis in our country.

## Figures and Tables

**Table 1:** Demographic, anatomical-clinical, and histopathological characteristics of the analyzed cases.

Baseline Characteristics	All Groups (*n* = 2443)		*p*-Value
	Study Group (*n* = 1780)	Control Group (*n* = 663)	
Demographic characteristics			
Age in years, mean ± SD	61.3 ± 11.3	60.5 ± 11.9	0.004
<60 years, *n* (%) ≥60 years, *n* (%)	762 (42.8) 1018 (57.2)	339 (51.1) 324 (48.9)	0.014
Gender, male/female, *n* (%)	1492/288 (83.8/16.2)	399/264 (60.2/39.8)	<0.001
Tobacco use (at presentation) (Yes/No), *n* (%)	1473/307 (82.7/17.3)	412/251 (62.1/37.9)	0.002
Alcohol abuse or dependence (Yes/No), *n* (%)	1285/495 (72.2/27.8)	104/559 (15.7/84.3)	<0.001
Poor oral hygiene (Yes/No), *n* (%)	564/1216 (31.7/68.3)	121(18.3/81.7)	0.004
Comorbidities (Yes/No), *n* (%)	761/1019 (42.8/57.2)	268/395 (40.4/59.6)	0.082
Anatomical-clinical and histopathological characteristics			
ANATOMIC SITE			
Lip	362 (20.3)	-	
Pelvilingual	374 (21)	-	
Tongue	412 (23.2)	-	
Floor of the mouth	221 (12.4)	-	
Gingival-alveolar	187 (10.5)	-	
Gingival-alveolar extended in Floor of the mouth and vice versa	91 (5.1)	-	
Soft palate	79 (4.4)	-	
Intermaxillary commissure with a jugal extension/toward the sidewall of the pharynx	54 (3)	-	
Clinical anatomical types—advanced stages			
Ulcero-vegetant	420 (23.6)	-	
Ulcero-destructive	396 (22.3)	-	
Infiltrative-diffuse	256 (14.4)	-	
Sclerosal form	127 (7.1)	-	
Lymphoganglionary Dissemination			
Metastatic lymphadenopathy	1321 (74.2)	-	
No dissemination	459 (25.8)	-	
Distribution of patients by clinical stage			
Stage I	221 (12.4)		
Stage II	651 (36.6)		
Stage III	487 (27.4)		
Stage IV	421 (23.7)		
Degree of differentiation			
well-differentiated	985 (55.3)	-	
moderately differentiated	598 (33.6)	-	
poorly differentiated	176 (9.9)	-	
Undifferentiated	21 (1.2)	-	

Continuous variables were expressed as mean ± standard deviation; categorical variables: number (%). ANOVA -test or Mann–Whitney U test for continuous variables. Pearson chi-square test.

**Table 2:** APC—Annual percentage change for regression slopes of patient age and incidence of oral mucosa carcinomas.

Segment	Age (Years)		APC	APC		Test Statistic (t)	*p*-Value
	Lower Endpoint	Upper Endpoint		Lower CI	Upper CI		
Slope 1	16	46	−2.8	−24.4	−7.1	−3.42	0.0012
Slope 2	46	49	9.6	6.7	10.4	9.38	0.0081
Slope 3	49	64	−2.4	−5.3	−1.6	−1.65	0.1239
Slope 4	64	74	4.6	1.4	6.9	11.59	0.0108
Slope 5	74	80	−8.8	−9.1	−5.4	−7.36	0.00251
Slope 6	80	91	8.1	6.4	14.2	12.91	0.0024

**Table 3:** Univariate and multivariate logistic regression for predicting the occurrence of carcinomas of the oral mucosa.

Logistic Regression	Odds Ratio (95%CI)	SE	*p*-Value
**Univariate analysis**			
Age ≥ 60 years (reference: age < 60)	1.95 (1.38–2.59)	0.011	0.003
Gender, male (reference: female)	4.43 (3.84–5.80)	0.152	<0.001
Tobacco use (at presentation) (Yes)	2.46 (1.15–3.63)	0.024	0.001
Alcohol abuse or dependence (Yes)	2.79 (1.27–4.05)	0.211	0.002
Poor oral hygiene (Yes)	1.19 (0.87–2.99)	0.096	0.083
Comorbidities (Yes)	1.24 (0.97–6.14)	0.084	0.076
**Multivariate analysis, Method: Enter**			
Age ≥ 60 years (reference: age < 60)	1.48 (1.22–3.19)	0.054	0.009
Gender, male (reference: female)	3.61 (2.06–5.81)	0.043	0.036
Tobacco use (at presentation) (Yes)	2.97 (1.42–3.94)	0.021	0.001
Alcohol abuse or dependence (Yes)	1.85 (1.11–3.46)	0.045	0.015
Poor oral hygiene (Yes)	1.04 (0.45–5.13)	0.106	0.058
Comorbidities (Yes)	1.32 (0.47–2.54)	0.084	0.096

CI: Confidence interval; SE: Standard error.

**Table 4:** Life-table to evaluate the survival rate of carcinomas of the oral mucosa vs. treatment type.

Time Limit *(Moment of Evaluation)* (Months)	% Cumulative Surviving RT + CT	% Cumulative Surviving S + RT + CT	% Cumulative Surviving S + RT	% Cumulative Surviving RT
**5**	100	100.	100.0000	100.0000
**7**	100.	100	83.3333	96.4286
**9**	100	100	83.3333	89.2857
**11**	100	66.7	83.3333	78.3528
**13**	100	66.6667	83.3333	65.9813
**15**	85.7	66.6667	83.3333	56.5554
**17**	85.7	66.6667	83.3333	51.8424
**19**	54.5	66.6667	83.3333	51.8424
**21**	54.5	66.6667	83.3333	51.8424
**24**	54.5	66.6667	83.3333	51.8424

Log-rank Test: *p* = 0.000914.

**Table 5:** Event-free survival (EFS) in carcinomas of the oral mucosa.

Parameters	*N*	EFS *n* (%)	^†^ EFS: Kaplan–Meier Method Cumulative Proportion Surviving: 24 Months		*p*-Value
			Estimate	Std. Error	
Gender, male	56	8 (14.3%)	83.2%	0.057	<0.001
Age ≥ 60 years	24	14 (58.3%)	37.7%	0.031	0.292
Tobacco use	119	28 (23.5%)	71.1%	0.075	0.113
**Anatomical-clinical forms**					0.109
Ulcero-vegetant	34	13 (38.2%)	54.7%	0.042	
Ulcero- destructive	21	5 (23.8%)	71.9%	0.074	
Infiltrative-diffuse	14	5 (35.7%)	46.4%	0.051	
Sclerotic shape	6	3 (50%)	50%	0.104	
**Lymph node dissemination**					
Metastatic lymphadenopathy	33	10 (30.3%)	69.1%	0.040	0.327
No metastatic lymphadenopathy	31	10 (32.3%	59.2%	0.041	
**Clinical Stage**					0.159
Stage I	81	17 (20.9%)	75.6%	0.029	
Stage II	44	14 (31.8%)	57.2%	0.036	
Stage III	61	11 (18.1%)	72.6%	0.037	
Stage IV	107	25 (23.4%)	70.8%	0.018	
**Degree of differentiation**					0.327
well-differentiated	119	28 (23.5%)	71.1%	0.075	
moderately differentiated		6 (33.3%)	64.9%	0.046	
poorly differentiated		24 (29.6%)	62.7%	0.022	
Undifferentiated		7 (15.9%)	81.6%	0.049	

^†^ Kaplan–Meier method—log-rank test; EFS—event-free survival.

## Data Availability

Not applicable.
